# Evaluation of Chemical Composition, Acaricidal, and Repellent Activities of *Artemisia vulgaris* L. (Asteraceae) Essential Oil Against Gall Mite *Aceria pongamiae* Keifer (Acarina: Eriophyidae)

**DOI:** 10.3390/molecules30163326

**Published:** 2025-08-08

**Authors:** Maneesha Kunnathattil, Naduvilthara U. Visakh, Berin Pathrose, Thejass Punathil, Archana Elamkulam Ravindran, Arunaksharan Narayanankutty, Sangeetha G. Kaimal

**Affiliations:** 1 PG & Research Department of Zoology, Govt. College Madappally, Affiliated to University of Calicut, Calicut 673102, India; maneeshak2997@gmail.com (M.K.); thejassp@gmail.com (T.P.); 2 Department of Agricultural Entomology, College of Agriculture, Kerala Agricultural University, Thrissur 680656, India; visakh.nu@kau.in (N.U.V.); berin.pathrose@kau.in (B.P.); 3 Department of Botany, Providence Women’s College (Autonomous), Affiliated to University of Calicut, Kerala 673009, India; drarchanaer@providencecollegecalicut.ac.in; 4 PG & Research Department of Zoology, Division of Cell and Molecular Biology, St. Joseph’s College (Autonomous), Devagiri, Affiliated to University of Calicut, Kerala 673008, India; 5 Department of Zoology, Providence Women’s College (Autonomous), Affiliated to University of Calicut, Kerala 673009, India

**Keywords:** Eriophyidae, *Artemisia vulgaris*, essential oil, acaricidal, repellency

## Abstract

The increasing environmental and health concerns about synthetic pesticides have compelled researchers to investigate more sustainable, plant-based substitutes for pest management. Due to their unique modes of action and biodegradability, essential oils (EOs) represent effective bio-pesticides. This study examines the biological activities of *Artemisia vulgaris* (Asteraceae) EO (AVEO) against *Aceria pongamiae* Keifer (Eriophyidae), a destructive gall mite on *Pongamia pinnata* (Fabaceae), using fumigation, contact toxicity, and repellency assays for the first time. AVEO was isolated through hydro-distillation, yielding 0.86 ± 0.14% *v*/*w* and analyzed by GC-MS/MS, with camphor (28.94%), 4-tert-butylaniline (19.79%), *α*-pinene (6.61%), eucalyptol (6.39%), fenchol (6.03%), and camphene (5.43%) identified as major constituents. The bioassay of fumigation (0.25–1 µL/mL air) showed LC_50_ values decreased significantly from 1.29 (24 h) to 0.43 µL/mL air (72 h), while LC_50_ values of contact toxicity bioassay (2.50–10 µL/mL) declined from 37.37 to 4.56 µL/mL over the same period. Repellency reached 86.11% (Class V) at 0.1 µL/mL (72 h), indicating intense concentration and time-dependent efficacy. These results indicate AVEO’s potential as a green acaricide, highlighting potent fumigant, contact, and repellent activities against *A. pongamiae*, positioning it as an eco-friendly alternative to synthetic acaricides for sustainable pest control practices with reduced environmental degradation.

## 1. Introduction

Eriophyid mites (Acari: Eriophyidae) are among the smallest arthropods known and present considerable challenges in research because of their minute size of approximately 200 µm length [1,2]. Despite these, it has been possible to identify around 4800 species, many of which are economically important pests of agronomically valuable crops [3]. Their small size and high reproductive potential make them difficult to control [3,4]. In addition, eriophyid mites have an uneven global distribution, with a disproportionate number of species in temperate regions [5]. Moreover, these mites are highly host-specific, with 95% of species being strictly associated with a single host plant genus [6]. These phytophagous pests have developed an intricate relationship with their hosts, often causing specific deformities and pathologies specific to the mite and plant species. These include leaf rolling, gall formation, stem and flower deformations, etc., which cause substantial economic losses in the agricultural and forestry sectors [7].

*Aceria pongamiae* Keifer is a highly host-specific eriophyid mite, producing varying numbers of finger-like foliar galls on *Pongamia pinnata* (L.) Pierre [8]. *P. pinnata,* commonly known as Karanj or Indian beach tree, is a famous flowering and fruiting angiosperm belonging to the Fabaceae family [9]. Native to Indian forests, this medium-sized and glabrous tree has been utilized in traditional Indian medicine, particularly in Ayurveda and Siddha systems, for treating various ailments, including bronchitis, whooping cough, rheumatism, diarrhea, dyspepsia, gonorrhea, leprosy, and tumors [10,11,12,13,14]. Moreover, *P. pinnata* has been recognized for its antimicrobial and antibacterial properties [9,15], and its seeds have potential in biofuel production [16]. The gall formation caused by *A. pongamiae* significantly impairs the quality of *Pongamia* leaves, reducing the plant’s economic value. The galls, which vary in color from green to yellowish-green to brownish-green, are epiphyllous and stalked, with a hard, unilocular, and indehiscent structure. Each gall harbors hundreds of mites in various developmental stages. Histopathological changes in *P. pinnata* are closely linked to the galling lifestyle of *A. pongamiae*, as mite infestation affects the plant’s physiology and growth [8].

The primary method of controlling eriophyid pest mites has been the use of synthetic pesticides, supplemented with biological control measures involving predatory mites (Family: Phytoseiidae and Stigmaeidae) [17,18,19], entomopathogenic fungi (e.g., *Hirsutella thompsonii* Fisher) [20,21], and other pathogens. However, the persistent use of conventional chemical acaricides has resulted in environmental pollution, elimination of beneficial organisms like natural enemies and insect pollinators [22], and increased health risks to humans [23]. Additionally, uncontrolled use of pesticides leads to the development of pesticide resistance, which reduces their efficiency [24]. Therefore, developing natural and efficient alternatives to synthetic pesticides is necessary to mitigate such negative effects.

Botanicals or plant natural products, especially essential oils (EOs), have many benefits, such as biodegradability, availability, and effectiveness on specific targets without developing pest resistance due to their complex chemical composition [25,26,27,28,29]. While EOs are often considered safer alternatives to synthetic pesticides, it is important to note that some EOs or its compounds can exhibit toxicity to mammals at high concentrations; hence their safe use typically requires appropriate dilution and formulation [30]. As one of the most promising botanical pesticide alternatives [24,31], EOs account for 1% of the insecticide market share globally [32].

EOs exhibit multiple modes of action, causing diverse biological changes in target organisms by altering key neurological and physiological pathways. The active compounds of EOs, such as monoterpenes, can interfere with the nervous system by modulating neurotransmitter activity, including acetylcholinesterase (AChE) inhibition [33,34,35], modifying γ-aminobutyric acid (GABA) receptors [36,37], and interfering with octopamine/tyramine receptors [38,39], leading to paralysis or death. EOs disturb the hormonal regulation and metabolic pathways for growth, survival, and reproduction [40,41]. They also affect the respiratory system through fumigation, disrupting cellular respiration [42,43]. They can be repellents by interfering with olfactory receptors [44], anti-feedants by altering taste receptor sensitivity [45], and contact toxins by rapid penetration via cuticle [46,47]. Biological impacts of EOs include cytotoxicity [48], phytotoxicity [49], mutagenicity and carcinogenicity [50], and neurotoxicity [51,52,53]. Interestingly, it has been determined that EOs also exhibit anti-mutagenic properties; for example, *Lavandula officinalis* [54] and *Eucalyptus globulus* [55] EOs have shown protective effects against mutagen-induced genotoxicity. Studies have indicated that whole EOs are typically more potent than individual components. Some constituents are known to facilitate cellular accumulation and absorption of toxic compounds [56,57].

Researchers have recently focused on using EOs as control agents for insect pests in greenhouses and stored products [58,59,60,61,62,63,64,65,66,67,68] because they have compatibility with natural predators, short residual effects, and show low toxicity towards human beings [69,70]. Many works have studied the application of EOs in managing various economically important pest mites [71,72,73,74,75,76,77,78,79,80,81,82,83]. The genus *Artemisia* is commonly known as wormwood or sagebrush, one of the largest and most widely distributed genera within the Asteraceae family, which is notable for its high EO content and favorable volatility characteristics, making it an important family for plant-based pesticide research [84,85]. EOs and extracts of many species from Asteraceae have been shown to exhibit fungicidal, insecticidal and acaricidal properties by many researchers [52,61,86,87,88,89,90,91,92,93,94]. Specifically, the acaricidal potential of EOs derived from several *Artemisia* species (*A. vulgaris*, *A. campestris*, *A. judaica*, *A. herba alba*, *A. annua*, *A. absinthium,* and *A. sieberi*) has been explored in various studies [93,94,95,96,97,98,99].

Although the insecticidal and acaricidal activities of plant EOs have been well-documented, a few studies have investigated their toxicity against eriophyid mites [76,82,100,101,102]. Notably, no research has been conducted on the toxicity of *Artemisia* EOs on eriophyid mites. Therefore, this study aims to investigate the chemical composition, acaricidal, and repellent activities of common mugwort, *A. vulgaris* EO (AVEO), against eriophyid mite *A. pongamiae* using laboratory bioassays for the first time.

## 2. Results

### 2.1. Percentage Yield and Analysis of Chemical Composition of AVEO by GC-MS/MS

**Figure 1:**
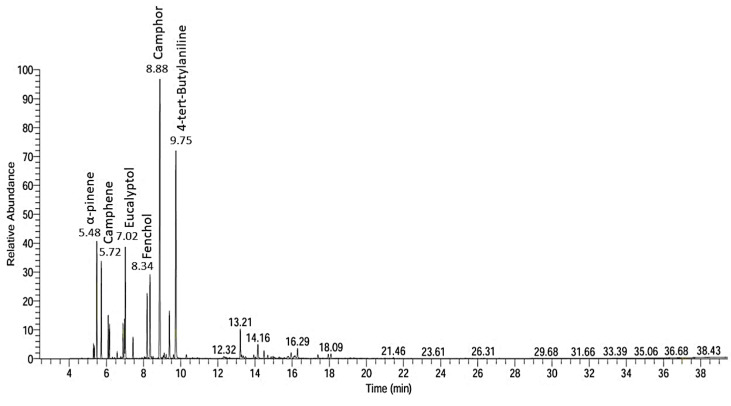
Chromatogram of GC–MS analysis of AVEO.

The EO extracted from *A. vulgaris* using hydro-distillation yielded 0.86 ± 0.14 (*v*/*w*). A total of 24 chemical constituents, representing 99.57% of the total EO composition, were identified through GC-MS/MS analysis. The corresponding chromatogram is shown in Figure 1, and the retention time and percentage composition of the identified constituents are listed in Table 1. The major constituents in AVEO belonged to the class of oxygenated monoterpenes (45.20%), followed by aromatics (21.88%) and monoterpene hydrocarbons (21.29%). Among these, camphor (28.94%), 4-tert-butylaniline (19.79%), α-pinene (6.61%), eucalyptol (6.39%), fenchol (6.03%), and camphene (5.43%) are primary compounds.

### 2.2. Evaluation of the Toxicity of AVEO Against A. pongamiae

#### 2.2.1. Fumigant Toxicity

**Figure 2:**
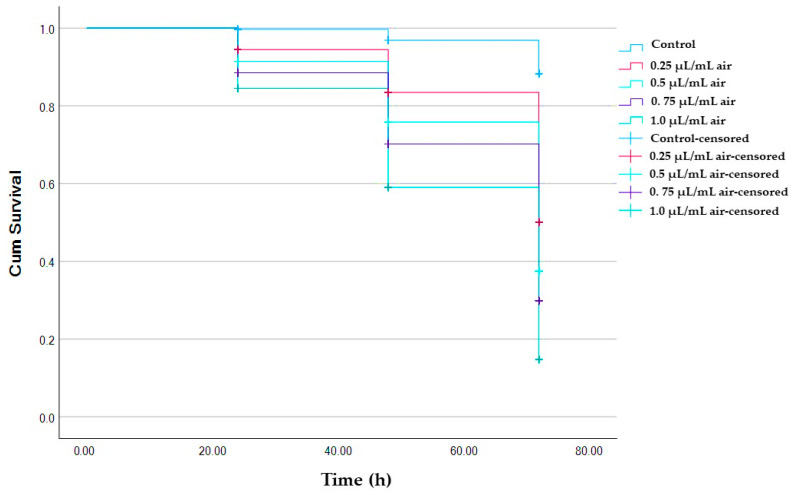
Kaplan–Meier survival curves showing fumigant toxicity of AVEO against *A. pongamiae* after 24, 48, and 72 h of exposure.

The results of the fumigant activity of AVEO against *A. pongamiae* demonstrated that various concentrations of AVEO (0.25–1 µL/mL air) elicited significant mortality at 48 and 72 h post-exposure. Even at the lowest concentration (0.25 µL/mL air), AVEO exhibited notable toxicity, with mortality rates of 23.3% and 40% after 48 and 72 h, respectively. Exposure to AVEO at a higher concentration (1 µL/mL air) resulted in substantially increased mortality rates, with values of 46.59, 60.23, and 75% after 24, 48, and 72 h, respectively (Table S1, Figure S1). Probit analysis revealed a significant decrease in the lethal concentration (LC_50_) of AVEO with extended exposure periods, with values of 1.29, 0.84, and 0.43 µL/mL air after 24, 48, and 72 h, respectively (Table 2). Survival analysis using Kaplan–Meier method revealed significant differences in the survival curves of mites exposed to different concentrations of AVEO over time Log Rank (Mantel–Cox) test (*X*^2^ = 182.17; df = 4; *p* < 0.001) (Tables S2–S4). These findings suggest that AVEO exhibits concentration and time-dependent fumigant activity against *A. pongamiae*, with increased mortality rates observed at higher concentrations and longer exposure times (Figure 2).

Table 2 presents the percentage of mortality of adult *A. pongamiae* after exposure to five various concentrations (0.25, 0.5, 0.75, and 1 µL/mL air) of AVEO in the fumigant toxicity test.

#### 2.2.2. Contact Toxicity

**Figure 3:**
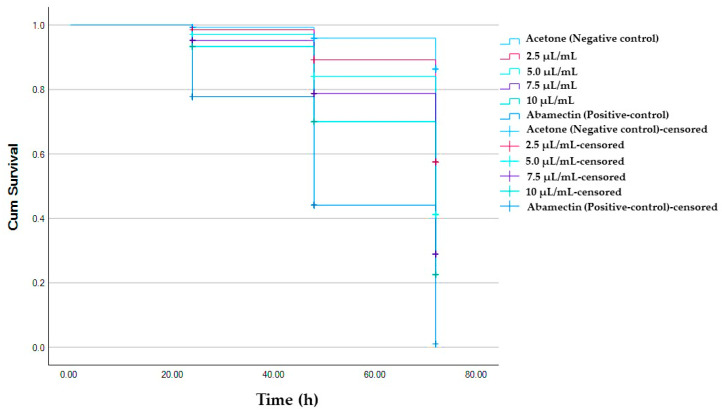
Kaplan–Meier survival curves illustrating contact toxicity of AVEO against *A. pongamiae* after 24, 48, and 72 h of exposure.

The leaf disc painting method revealed that AVEO exhibited significant toxicity to adult *A*. *pongamiae*, with mortality rates of 20, 50, and 67.8% after 24, 48, and 72 h, respectively, when exposed to higher concentrations of 10 µL/mL of AVEO, while abamectin resulted in 99.17% mortality after 72 h exposure (Table S5, Figure S2). Probit analysis showed that the LC_50_ values for AVEO against adult *A*. *pongamiae* were 37.37, 12.14, and 4.56 µL/mL after 24, 48 and 72 h, respectively. The Kaplan–Meier survival analysis, followed by the Log Rank test, showed significant contact toxicity of AVEO on mite survival (*X*^2^ = 394.20; df = 5; *p* < 0.001) (Tables S6–S8), with higher concentration leading to reduced survival rates (Figure 3). Table 3 demonstrates the percentage mortality of adult *A*. *pongamiae* exposed to various AVEO concentrations (2.50 to 10 µL/mL).

#### 2.2.3. Repellent Activity

The repellent activity of AVEO against *A. pongamiae* was evaluated, revealing a strong concentration-dependent response (Table 4). The various concentrations of AVEO and exposure times demonstrated substantial repellent activity, with repellency rates (PR) ranging from 38.21% (class II) at the lowest concentration (0.025 µL/mL) to 86.11% (class V) at the highest concentration (0.1 µL/mL) after 72 h of exposure at a 20 µL dose. Notably, PR values exceeded 50% (class III) at 0.05 µL/mL and 81.48% (class IV) at 0.075 µL/mL after 72 h exposure (Table 4).

## 3. Discussion

The present study evaluates the acaricidal and repellent properties of AVEO against the eriophyid gall mite *A. pongamiae*. Our findings demonstrate that AVEO exhibits significant fumigant toxicity, contact toxicity, and repellency, highlighting its potential as a botanical acaricide for sustainable pest management.

In this study, the hydro-distillation of AVEO yielded 0.86 ± 0.14% (*v*/*w*), which is consistent with the study of Singh et al. [103], who reported an AVEO content of 0.75% (*w*/*v*) from specimens in India and is inconsistent with other reported yields from differing geographical regions and plant developmental stages. Trinh et al. [104] recorded a lower yield of 0.26% from fresh aerial parts of *A. vulgaris* in Tien Giang province, Vietnam. Likewise, Malik et al. [105] also reported a yield of 0.5% (*v*/*v*) from Brazilian *A. vulgaris*. Such discrepancies indicate that the geographical and climate conditions are essential determinants of EO production. Importantly, Han et al. [106] reported a yield of 0.31% (*v*/*w*) from dried aboveground flowering plant materials, demonstrating the influence of plant phenology on EO accumulation. Furthermore, Sharma and Adhikari [107] observed that altitude plays a crucial role in EO yield, with *A. vulgaris* collected from different regions of Nepal exhibiting varying yields (0.3% in Chitwan and Gorkha, 0.2% in Kathmandu). The higher altitude of Kathmandu (1324 m) resulted in a reduced EO yield compared to lower-altitude regions, reinforcing the impact of environmental factors on secondary metabolite biosynthesis. The comparatively higher yield obtained in our study may be due to differences in plant maturity, soil composition, post-harvest handling, or distillation efficiency. These findings align with existing literature emphasizing that intrinsic (genetic) and extrinsic (ecological) factors collectively determine EO composition and yield [108].

The chemical composition of AVEO obtained in the present study was characterized by a predominance of oxygenated monoterpenes (45.20%), followed by aromatics (21.88%) and monoterpene hydrocarbons (21.29%). The major constituents included camphor (28.94%), 4-tert-butylaniline (19.79%), *α*-pinene (6.61%), eucalyptol (6.39%), fenchol (6.03%), and camphene (5.43%). This compositional profile exhibits similarities and notable differences compared with previous reports on AVEO from different geographical regions, highlighting the influence of genetic, environmental, and methodological factors on secondary metabolite production. For instance, Trinh et al. [104] identified 1,8-cineole (24.25%), *α*-pinene (10.57%), *β*-caryophyllene (7.10%), borneol (8.89%), camphor (6.87%), and *δ*-elemene (6.05%) as major components in Vietnamese AVEO obtained via hydro-distillation, whereas headspace analysis of the same sample revealed higher proportions of camphor (27.16%) and 2-methylbutanal (20.94%). These variations between hydro-distillation and headspace methods suggest that extraction techniques significantly impact the detected volatile profile, with headspace favoring more volatile and low-molecular-weight compounds. Similarly, Malik et al. [105] reported a distinct chemotype dominated by sesquiterpenes, particularly caryophyllene (37.45%) and germacrene-D (16.17%), which contrasts with the monoterpene-rich composition observed in our study.

Phenological stage and post-harvest processing also play critical roles in EO composition. Han et al. [106] analyzed AVEO from flowering-stage plants and found (*Z*)-sabinol (15.18%), trans-sabinyl acetate (11.13%), and eucalyptol (10.92%) as predominant compounds, differing from both our results and other studies. The observed variability in AVEO composition across studies may also stem from genetic differences among regional populations. For example, Munda et al. [109] identified 1,8-cineole (16.76%) and camphor (11.94%) as major components in Indian *A. vulgaris*, differing with our findings but aligning partially with secondary constituents like borneol (8.06%). Additionally, Houti et al. [110] demonstrated that seasonal climatic variations such as spring rainfall and summer hydric stress alter the proportions of oxygenated monoterpenes such as *β*-thujone in *Artemisia* species, further underscoring the environmental modulation of EO’s composition.

The present study demonstrated that AVEO exhibits significant fumigant toxicity against *A. pongamiae* in a concentration- and time-dependent manner. At 1 µL/mL air, AVEO induced 75% mortality after 72 h, with the LC_50_ decreasing from 1.29 µL/mL air (24 h) to 0.43 µL/mL air (72 h). These results align with prior studies on eriophyid mites, where EOs have shown promising acaricidal properties. For instance, Eucalyptus EO exhibited significant fumigant toxicity against *A. pongamiae* (LC_50_ = 1.01% at 24 h) [82]. Notably, *A. vulgaris* EO exhibited dose-dependent fumigant toxicity against stored-product pests such as *Tribolium castaneum* (LC_50_ = 279.86 µL/L air) and *Callosobruchus maculatus* (LC_50_ = 52.47 µL/L air) [111]. Similarly, *A. absinthium* EO exhibited significant fumigant activity against *Tetranychus urticae* [112], reinforcing the potential of *Artemisia*-derived EOs in pest management.

The contact toxicity assays in this study further validated the acaricidal potency of AVEO, with mortality rates reaching 67.8% at 10 µL/mL after 72 h. The LC_50_ values decreased with prolonged exposure (37.37 µL/mL at 24 h to 4.56 µL/mL at 72 h), suggesting concentration-and time-dependent efficacy, in line with the study by Kunnathattil et al. [82], who reported that black pepper EO demonstrated superior contact toxicity (LC_50_ = 0.92, 0.68 and 0.46% at 24, 48, and 72 h) against *A. pongamiae*. Comparable trends were observed in other studies; for example, *A. campestris*, *A. judaica,* and *A. herba alba* EOs exhibited high contact toxicity against the honey bee mite *Varroa destructor*, with selectivity ratios ranging from 5.62 to 10.77 [94]. Additionally, *Artemisia* EOs have demonstrated ovicidal and adulticidal effects against *Ectomyelois ceratoniae* [113], highlighting their broad-spectrum bioactivity. The variability in LC_50_ values across studies may reflect differences in EO composition, target species, or experimental conditions. For example, Gao et al. [43] reported minimal time-dependent effects of AVEO on *T. castaneum* (LC_50_ = 4.77 to 6.14% over 72 h), contrasting with our findings for *A. pongamiae*. The effectiveness of abamectin as a contact toxicant was confirmed, with 99.17% mortality (LD_50_ = 1.26 µL/cm^2^) observed after 72 h exposure. These findings are consistent with previous studies that have demonstrated the efficacy of abamectin against various mite species, including *T. urticae* [114], *A. litchii* [115], and *Phyllocoptruta oleivora* [116]. The mode of action of abamectin, which involves disrupting the muscle and nervous system of insects and acarines through contact and ingestion [117,118,119], is likely responsible for its effectiveness as a contact toxicant.

The efficacy of AVEO on eriophyid mites aligns with earlier reports on other botanicals. For instance, *Allium sativum* EO nano-emulsions showed high toxicity against *A. oleae* [101], *Citrus* EO has superior bioactivity against *A. guerreronis* [100], while *Thymus* EO effectively suppressed *Cisaberoptus kenyae* [102]. Furthermore, the acaricidal potency of *A. vulgaris* EO against *Bemisia tabaci* and *T. urticae* supports its potential as a broad-spectrum miticide [120]. However, the limited literature on botanical control of eriophyid mites [76,100] necessitates further research to optimize EO formulations and application methods.

This study provides the first reported evidence of the repellent activity of EOs against the eriophyid mite. AVEO exhibited a strong concentration-dependent repellent response, with repellency rates (PR) ranging from 38.21% (Class II) at 0.025 µL/mL to 86.11% (Class V) at 0.1 µL/mL after 72 h of exposure. PR values surpassed the 50% threshold (Class III) at 0.05 µL/mL and reached 81.48% (Class IV) at 0.075 µL/mL, indicating high efficacy at elevated concentrations. These findings align with prior studies on *Artemisia* EOs, which have demonstrated pronounced repellency against arthropod pests due to their volatile terpenoid constituents [121]. For instance, AVEO exhibited robust repellency against *T. castaneum* [122,123], while *A. sieberi* EO caused significant repellent effects on mites at varying doses over 48 h [99]. The observed repellency of AVEO may be attributed to its bioactive monoterpenes, which are also consistent with reports on *A. campestris* and *A. herba alba* EOs against *B. tabaci*, suggesting that these plant species could be valuable sources for developing natural pesticides to control destructive insect pests [97].

## 4. Materials and Methods

### 4.1. Collection and Stock Culture of Test Mite

Both galled and ungalled leaves of *P*. *pinnata* were obtained at Malaparamba, Calicut district, Kerala, India (11.2976° N, 75.8059° E). The study was conducted between April and October of 2024. Each leaf was examined under a stereo zoom trinocular research microscope (Magnus MSZ-TR, Olympus Opto Systems India Pvt. Ltd., Noida, India; 400× magnification) and galls were dissected to isolate adult *A*. *pongamiae*. Species identification was carried out based on morphological characteristics and confirmed by Dr. Sangeetha G Kaimal (Acarologist, Department of Zoology, Providence Women’s College (Autonomous), Calicut, India). The laboratory cultures of adult *A*. *pongamiae* were used in all bioassays by the leaf flotation technique [82].

### 4.2. Plant Material and Isolation of EO

Fresh leaves of *A. vulgaris* were gathered from the Kozhikode district in Kerala, India (11.2588° N, 75.7804° E). The leaves were shade-dried at room temperature for five days to reduce moisture content. A total 500 g of dried leaves was subjected to hydro-distillation using Clevenger apparatus for 4 h. After dehydration with anhydrous sodium sulfate, the extracted AVEO was weighed and stored in the amber-colored glass bottles at 4 °C until further use.

The EO yield was calculated as a percentage using the following formula:

Yield of oil (% *v*/*w*) = Volume of dried EO/Weight of dried samples × 100

### 4.3. GC-MS/MS Analysis of Chemical Constituents Present in AVEO

The chemical characterization of the AVEO was conducted using Gas Chromatography and Mass Spectrometry (GC-MS/MS) with the TSQ 8000 Evo device (Thermo Scientific, Waltham, MA, USA), which was equipped with an auto-sampler and a TG-1MS capillary column measuring 30 m × 0.25 mm × 0.25 µm. The oven temperature was programmed as follows: 50 °C for 1 min, 10 °C/min to 120 °C and then to 270 °C for 5 min at 5 °C/min. The injector temperature was set at 250 °C. The samples (0.1 μL) were injected with a split ratio of 1:200. Helium was used as the carrier gas at a flow rate of 1.0 mL/min. Mass spectra were scanned from 35 to 500 *m*/*z* with a dwell time of 0.2 ms [60]. The mass spectrometry data have been recorded and analyzed using Xcalibur 1.1 software. The percentage composition was established by examining the retention time and comparing the relative peak areas derived from the GC-MS/MS chromatogram with those in the standard NIST library. Additionally, a mixture of n-alkanes (C_7_–C_30_) was co-injected into the GC-MS column to determine the retention indices (RIs) of each chemical component under identical conditions. This approach enabled double-confirmation of chemical compound identification by combining RI matching with NIST library.

### 4.4. Toxicity Tests

#### 4.4.1. Fumigant Toxicity Test

To assess the toxicity of EOs as fumigants against *A*. *pongamiae*, a modified protocol of Kunnathattil et al. [82] was implemented. AVEO was diluted with HPLC-grade acetone in varying ratios to prepare different concentrations (0.25, 0.5, 0.75, and 1 µL/mL air). Using a fine brush, thirty adult *A*. *pongamiae* specimens were randomly selected and transferred onto the leaf disc of *P*. *pinnata* (1 × 1 cm), which was placed on Petri dishes (9.5 cm in diameter) lined with moist spongy pads. A filter paper disc (1.5 cm in diameter, Whatman No. 1) was affixed to the inner surface of the Petri plates. Each filter paper disc received 20 µL of the respective oil concentration using a micropipette, and all treatment groups were securely sealed with parafilm to prevent evaporation. A control group was established using a filter paper disc with 20 µL of acetone alone (the EO diluent) as the control, allowing complete evaporation before the assay to eliminate any potential fumigation effects. The experimental setup was designed to avoid direct contact between the mites and the filter paper disc [59]. To ensure accuracy, both the experimental and control groups have been replicated three times. Throughout the treatment groups, the temperature and relative humidity were kept at 22 ± 2 °C and 85 ± 2%, respectively. The mortality percentages were corrected using Abbott’s formula [124] after 24, 48, and 72 h, and LC_50_ values were worked out.

#### 4.4.2. Contact Toxicity Test

The contact toxicity of AVEO against *A*. *pongamiae* was evaluated using the leaf disc painting technique, which has been modified by Miresmailli et al. [125]. AVEO was diluted using the solvent DMSO (dimethyl sulfoxide) to prepare four distinct concentrations: 2.5, 5, 7.5, and 10 µL/mL. Each concentration of the AVEO was painted onto the leaf disc of *P*. *pinnata* (1 × 1 cm) set on moist spongy pads at the base of a Petri dish. Following a drying period of 5 min at room temperature, 30 adult mites were individually transferred to each treated leaf disc using a fine brush. Perforated plastic lids were placed on the Petri dishes to allow airflow and minimize the risk of fumigant toxicity. As a negative control, the same number of mites were maintained on DMSO-treated leaf discs. Additionally, chemical miticide abamectin (Abamectin 1.9% EC, EBS Antigo, 0.75 mL/L in water) was used as a positive control, under the same conditions. Three replicates were conducted for both the treatments and the controls. After 24, 48, and 72 h, all discs were examined under a stereo zoom trinocular research microscope. As previously noted, Abbott’s formula was applied to correct the mortality data, allowing for the calculation of LC_50_ values by plotting the mortality percentages against the concentrations of AVEO.

#### 4.4.3. Repellent Activity Assay

**Figure 4:**
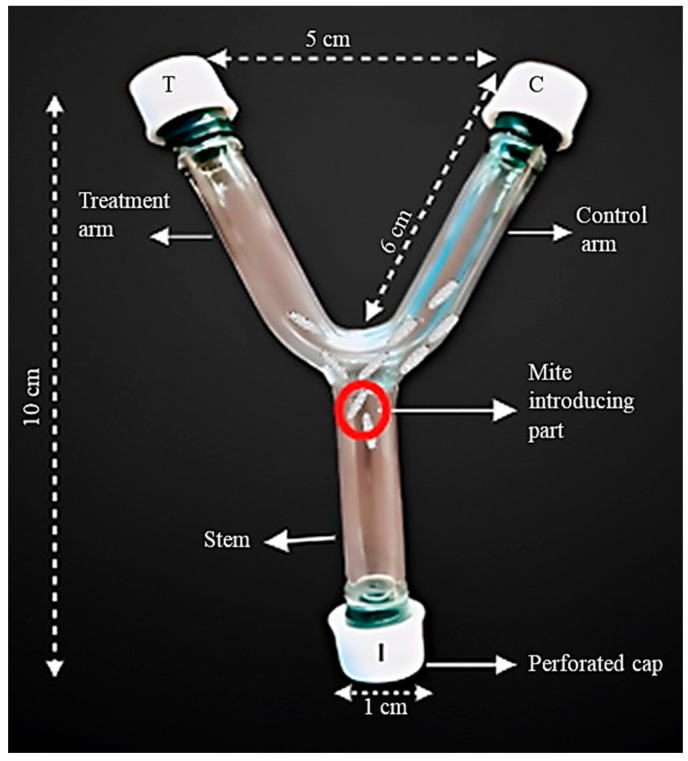
Experimental setup of Y-tube olfactometer bioassay, showing (T) treatment arm with filter paper disc treated with test concentrations of AVEO placed inside of Cap-T, (C) control arm with filter paper disc treated with acetone placed inside of Cap-C, and (I) introduction point for a fixed number of mites through the stem, closed with a perforated cap.

The repellency test was conducted according to Motazedian et al. [22], with minor modifications. Using a Y-tube olfactometer bioassay, the repellency effect was examined. A glass tube with a diameter of 1 cm, featuring a main arm or stem (5 cm length) and two other arms (6 cm length), one serving as the treatment (test) arm and the other as the control (Figure 4). The test solutions were prepared by diluting the AVEO in acetone to obtain concentrations of 0.025, 0.05, 0.075, and 0.1 µL/mL. A volume of 20 µL of each test concentration was applied to a filter paper disc (1 cm diameter, Whatman No. 1) and placed at the end of the treatment arm. In the control arm, a filter paper disc treated with 20 µL of acetone alone was placed. All solvent-treated filter paper discs were allowed to air dry before use to prevent fumigation effects.

A group of 15 adult *A*. *pongamiae* was introduced through the stem near the test area between the two connected arms. The upper end of the apparatus was sealed with a plastic cap and secured with parafilm, and the main arm was closed with a perforated plastic cap. After intervals of 24, 48, and 72 h, the number of mites in each arm was recorded, and three replications of the experiment were conducted for each concentration. The experimental design of the repellency test using the Y-tube olfactometer bioassay is depicted in Figure 4. The percentage of repellence (PR) was determined using the following formula;

PR = (NC − NT)/(NC + NT) × 100

where PR is the percentage of repellence (%), NC is the number of test mites in the control arm, and NT is the number of test mites in the treated arm.

The repellency was assessed and classified according to Visakh et al. [60]; Class 0 (0 to 0.1% PR, no or very weak repellent), Class I (0.2 to 10% PR, weak repellent), Class II (20.1 to 40% PR, repellent to some extend), Class III (40.1 to 60% PR, medium repellent), Class IV (60.1 to 80% PR, very good repellent), and Class V (80.1 to 100% PR, highly repellent).

### 4.5. Statistical Analysis

The data are presented as the mean ± SD from all experiments conducted. A comprehensive evaluation of the data was performed using analysis of variance (ANOVA), followed by Dunnett’s post hoc test and the least significant difference (LSD) test. Survival data were analyzed using Kaplan–Meier curves with log-rank tests. All statistical analyses were performed using the SPSS statistical software (IBM SPSS Statistics version 30).

## 5. Conclusions

The present study demonstrates the significant acaricidal and repellent potential of AVEO against *A. pongamiae*, a destructive eriophyid gall mite affecting *P. pinnata*. AVEO, rich in bioactive compounds such as camphor, 4-tert-butylaniline, *α*-pinene, eucalyptol, fenchol, and camphene, exhibited strong fumigant, contact toxicity, and repellent activities in a concentration–time-dependent manner. The fumigant bioassay revealed high toxicity with progressively decreasing LC_50_ values, while contact toxicity also showed increasing efficacy over time. Additionally, AVEO displayed potent repellency at the tested concentrations. Our findings highlight AVEO as a promising botanical acaricide for managing eriophyid mites, with significant fumigant, contact, and repellent activities. However, further research is needed to optimize EO formulations, assess field efficacy, and evaluate non-target effects. Given the limited literature on botanical control of eriophyid mites, this study provides a foundation for developing eco-friendly pest management strategies. Future studies should explore synergistic interactions with other plant-derived compounds and the development of stable nano-formulations to enhance AVEO’s bioactivity and persistence.

## Figures and Tables

**Table 1:** Percentage composition of chemical constituents in AVEO identified by GC-MS/MS based on RI and MS data.

Sl. No.	RT ^a^	Compound Name	Chemical Formula	RI ^b^	RI ^c^	Area %
1.	5.30	Tricyclene	C_10_H_16_	903	906	0.77
2.	5.36	β-Thujene	C_10_H_16_	921	920	0.68
3.	5.48	α-Pinene	C_10_H_16_	932	939	6.61
4.	5.72	Camphene	C_10_H_16_	960	959	5.43
5.	6.09	α-Phellandrene	C_10_H_16_	984	985	2.36
6.	6.16	β-Terpinen	C_10_H_16_	985	988	1.90
7.	6.89	o-Cymene	C_10_H_14_	1024	1022	2.09
8.	6.97	D-Sylvestrene	C_10_H_16_	1029	1027	2.32
9.	7.02	Eucalyptol	C_10_H_18_O	1033	1035	6.39
10.	7.43	γ-Terpinene	C_10_H_16_	1065	1062	1.22
11.	8.19	Thujone	C_10_H_16_O	1073	1079	3.84
12.	8.34	Fenchol	C_10_H_18_O	1087	1090	6.03
13.	8.88	Camphor	C_10_H_16_O	1140	1145	28.94
14.	9.39	L-Terpinen-4-ol	C_10_H_18_O	1185	1182	3.33
15.	9.75	4-Tert-butylaniline	C_10_H_15_N	1279	1270	19.79
16.	13.21	α-Copaene	C_15_H_24_	1376	1378	2.40
17.	14.16	Caryophyllene	C_15_H_24_	1414	1411	1.30
18.	14.49	α-Guaiene	C_15_H_24_	1426	1424	0.67
19.	15.80	α-Muurolene	C_15_H_24_	1495	1499	0.48
20.	15.95	δ-Guaijene	C_15_H_24_	1508	1506	0.65
21.	16.29	δ-Cadinene	C_15_H_24_	1520	1522	0.99
22.	17.39	Hexyl benzoate	C_13_H_18_O_2_	1580	1581	0.45
23.	17.95	Carotol	C_15_H_26_O	1595	1593	0.44
24.	18.09	Cedrol	C_15_H_26_O	1604	1609	0.49
		Monoterpene hydrocarbons (%)				21.29
		Oxygenated monoterpenes (%)				45.20
		Sesquiterpene hydrocarbons (%)				6.49
		Oxygenated Sequiterpene (%)				0.93
		Aromatics (%)				21.88
		Others (%)				3.78
		Total compounds (%)				99.57

**^a^** Retention time. **^b^** Retention index determined through retention times of n-alkanes (C_7_–C_30_) series. **^c^** Retention index from NIST library.

**Table 2:** Toxicity of AVEO in fumigant toxicity test against *A. pongamiae* after 24, 48, and 72 h exposure.

Time	Concentration (µL/mL Air)	Mean Mortality ± SD	%Mean Mortality ± SD	LC_50_ * (µL/mL)	Slope	F Value	*p* Value
24 h	Control	0.33 ± 0.58	24.94 ± 17.29 ^a^	1.29 (0.96–2.4)	1.41	95.29	<0.01
	0.25	5.00 ± 1.00 ^S^					
	0.50	7.33 ± 1.15 ^S^					
	0.75	10.00 ± 1.00 ^S^					
	1.00	13.67 ± 0.58 ^S^					
48 h	Control	1.67 ± 1.53	32.92 ± 20.37 ^a^	0.84 (0.68–1.17)	1.50	105.40	<0.01
	0.25	7.00 ± 1.00 ^S^					
	0.50	9.67 ± 0.58 ^S^					
	0.75	12.00 ± 1.00 ^S^					
	1.00	17.67 ± 0.58 ^S^					
72 h	Control	2.67 ± 0.58	46.38 ± 24.53 ^a^	0.43 (0.32–0.53)	1.39	108.52	<0.01
	0.25	12.00 ± 1.00 ^S^					
	0.50	14.33 ± 1.53 ^S^					
	0.75	16.67 ± 0.57 ^S^					
	1.00	22.00 ± 1.73 ^S^					
F = 1.339; *p* = 0.29							

^S^: significantly different from negative control at *p* < 0.05; % Mean mortality ± SD followed by letter ^a^ are non-significant pairs in least significant difference and Dunnett’s post hoc test. * LC _50_ of (upper limit-lower limit) 95% CL interval.

**Table 3:** Toxicity of AVEO in contact toxicity test against *A. pongamiae* after 24, 48, and 72 h exposure.

Time	Concentration (µL/mL)	Mean Mortality ± SD	%Mean Mortality ± SD	LC_50_ * (µL/mL)	Slope	F Value	*p* Value
24 h	Negative control	0.67 ± 0.58	10.22 ± 7.47 ^b^	37.37 (19.38–337.69)	1.45	22.60	<0.01
	2.50	1.33 ± 1.15 ^NS^					
	5.00	2.67 ± 0.58 ^S^					
	7.50	4.67 ± 0.58 ^S^					
	10.00	6.00 ± 1.00 ^S^					
	Positive control	20 ± 4.32 ^S^		LD_50_ = 5.59 (3.50–7.10) µL/cm^2^	1.51		
48 h	Negative control	2.00 ± 1.73	27.33 ± 16.30 ^b,c^	12.14 (9.13–22.35)	1.36	24.67	<0.01
	2.50	5.67 ± 1.58 ^S^					
	5.00	8.00 ± 2.00 ^S^					
	7.50	10.33 ± 1.53 ^S^					
	10.00	15.00 ± 1.73 ^S^					
	Positive control	27 ± 3.83 ^S^		LD_50_ = 2.23 (0.8–3.51) µL/cm^2^	1.84		
72 h	Negative control	3.00 ± 0.00	45.66 ± 23.51 ^c^	4.56 (3.47–5.59)	1.42	186.04	<0.01
	2.50	10.67 ± 1.53 ^S^					
	5.00	15.33 ± 0.58 ^S^					
	7.50	19.00 ± 1.00 ^S^					
	10.00	20.33 ± 0.58 ^S^					
	Positive control	29.75 ± 0.5 ^S^		LD_50_ = 1.26 (0.14–2.51) µL/cm^2^	1.58		
F = 5.391; *p* = 0.021							

^S^ significantly different from negative control; ^NS^ non-significant difference with control at *p* < 0.05; %Mean mortality ± SD followed by same letters (^b,c^) are non-significant pairs in least significant difference and Dunnett’s post hoc test. * LC _50_ of (upper limit-lower limit) 95% CL interval.

**Table 4:** Repellence of AVEO against *A. pongamiae* after 24, 48, and 72 h.

Concentration (µL/mL)	Repellence Percentage of the Treatment After			Mean Repellence Percent ± SD	Class
	24 h	48 h	72 h		
0.025	33.33 ± 0.00 ^d^	20 ± 0.00 ^e^	38.21 ± 4.76 ^g^	30.51 ± 9.43 ^i^	II
0.05	26.67 ± 23.09 ^d^	46.10 ± 3.62 ^e^	50 ± 0.00 ^g^	40.92 ± 12.50 ^i^	III
0.075	42.06 ± 8.36 ^d^	77.14 ± 20.20 ^f^	81.48 ± 16.97 ^h^	66.90 ± 21.62 ^j^	IV
0.1	75.56 ± 21.43 ^d^	82.22 ± 16.78 ^f^	86.11 ± 12.72 ^h^	81.30 ± 5.34 ^j^	V
F value	5.32	14.06	14.00	8.81	
*p* value	0.026	0.001	0.002	0.006	

Repellence percent and mean repellence percent ±SD within same column followed by same letters (^d–j^) are non-significant pairs in least significant difference and Dunnett’s post hoc test (*p* < 0.05).

## Data Availability

The data that support the findings of this study are available from the corresponding author upon reasonable request.

## Supplementary Materials

The following supporting information can be downloaded at: https://www.mdpi.com/article/10.3390/molecules30163326/s1, Figure S1: Mortality of *A. pongamiae* in fumigant toxicity test after 24, 48, and 72 h exposure of AVEO. Same letters (a, b, and c) indicate non-significant pairs; Figure S2: Mortality of *A. pongamiae* in contact toxicity test after 24, 48, and 72 h exposure of AVEO. Same letters (a, b, c, and d) indicate non-significant pairs; Table S1: Percentage mortality of *A. pongamiae* on fumigant toxicity test after 24, 48, and 72 h exposure of AVEO; Table S2: Case processing summary of fumigant toxicity test using Kaplan–Meier survival analysis; Table S3: Means and medians for survival time in fumigant toxicity test using Kaplan–Meier survival analysis; Table S4: Overall comparisons of survival of *A. pongamiae* exposed to different concentrations of AVEO over time Log Rank (Mantel–Cox) test after 24, 48, and 72 h in fumigant toxicity test; Table S5: Percentage mortality of *A. pongamiae* in contact toxicity test after 24, 48, and 72 h. exposure of AVEO; Table S6: Case processing summary of contact toxicity test using Kaplan–Meier survival analysis; Table S7: Means and medians for survival time in contact toxicity test using Kaplan–Meier survival analysis; Table S8: Overall comparisons of survival of *A. pongamiae* exposed to different concentrations of AVEO over time Log Rank (Mantel–Cox) test after 24, 48, and 72 h in contact toxicity test.

## Abbreviations

The following abbreviations are used in this manuscript:

EO	Essential oil
AVEO	*Artemisia vulgaris* essential oil
DMSO	Dimethyl sulfoxide
GC-MS/MS	Gas Chromatography and Mass Spectrometry
PR	Percentage of repellence
