# Supplementary material for: Evaluation of Chemical Composition, Acaricidal, and Repellent Activities of Artemisia vulgaris L. (Asteraceae) Essential Oil Against Gall Mite Aceria pongamiae Keifer (Acarina: Eriophyidae)
Source: Molecules. 2025 Aug 8;30(16):3326. doi: 10.3390/molecules30163326 (PMC12388139; doi:10.3390/molecules30163326)
Supplement: Supplementary file 1 [file molecules-30-03326-s001.zip › molecules-3780984-supplementary.pdf]

Evaluation of Chemical Composition, Acaricidal, and Repellent Activities of *Artemisia vulgaris* L. (Asteraceae) Essential Oil Against Gall Mite *Aceria pongamiae* Keifer (Acarina: *Eriophyidae*)

SUPPLEMENTARY MATERIALS

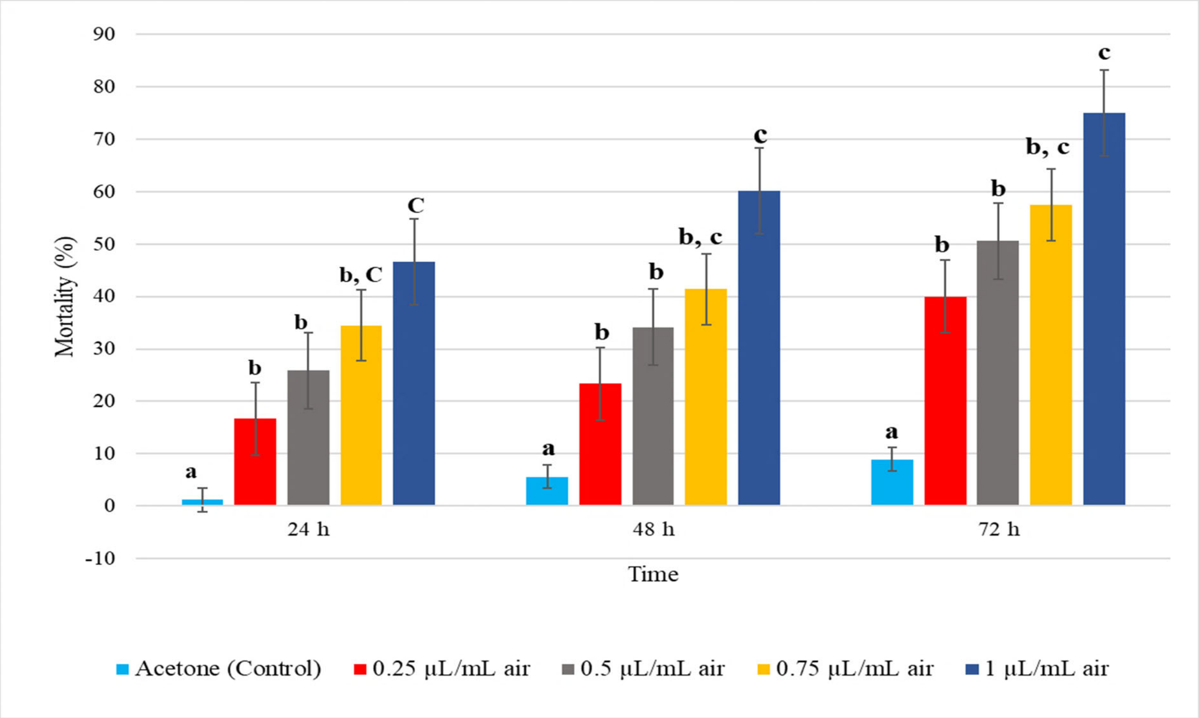

Supplementary Figure S1. Mortality of *Aceria pongamiae* in fumigant toxicity test after 24, 48, and 72 h. exposure of AVEO. Same letters (a, b, and c) indicate non-significant pairs

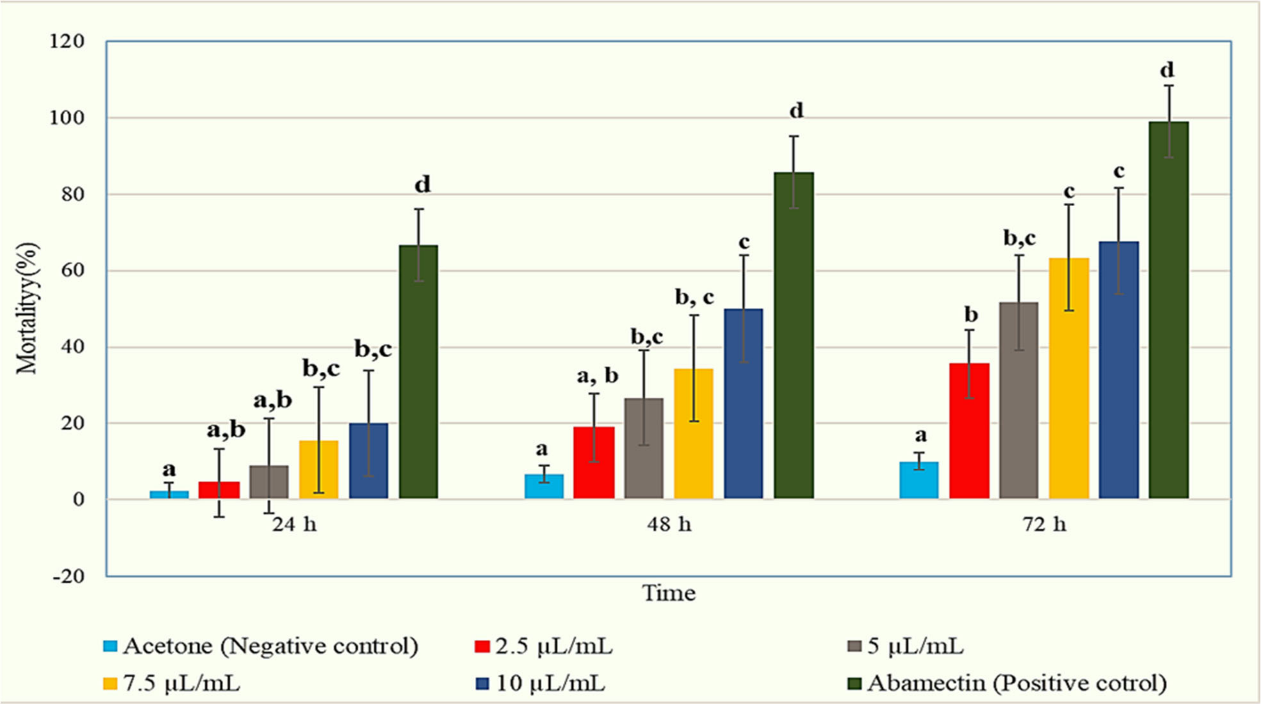

Supplementary Figure S2. Mortality of *A. pongamiae* in contact toxicity test after 24, 48, and 72 h. exposure of AVEO. Same letters (a, b, c, and d) indicate non-significant pairs.

Supplementary Table S1. Percentage mortality of *A. pongamiae* on fumigant toxicity test after 24, 48, and 72 h. exposure of AVEO.

Concentration	24 h	48 h	72 h
Acetone (Control)	1.11	5.56	8.89
0.25 µL/mL air	16.667	23.33	40
0.5 µL/mL air	25.88	34.12	50.59
0.75 µL/mL air	34.48	41.38	57.47
1 µL/mL air	46.59	60.27	75

Supplementary Table S2. Case processing summary of fumigant toxicity test using Kaplan-Meier survival analysis

Group	Total N	N of Events	Censored	
			N	Percent
1.00	270	14	256	94.8%
2.00	270	72	198	73.3%
3.00	255	94	161	63.1%
4.00	261	116	145	55.6%
5.00	264	160	104	39.4%
Overall	1320	456	864	65.5%

Group 1= Acetone (Control); Group 2= 0.25 µL/mL air; Group 3= 0.50 µL/mL air; Group 4= 0.75 µL/mL air; Group 5= 1.0 µL/mL air.

Supplementary Table S3. Means and medians for survival time in fumigant toxicity test using Kaplan-Meier survival analysis

Group	Mean[a]				Median		
	Estimate	Std. Error	95% Confidence Interval		Estimate	Std. Error	95% Confidence Interval
			Lower Bound	Upper Bound			Lower Bound
1.00	71.158	.354	70.464	71.852	.	.	.
2.00	66.689	.837	65.048	68.330	.	.	.
3.00	64.118	1.003	62.151	66.085	72.000	2.736	66.637
4.00	62.088	1.076	59.979	64.197	72.000	2.351	67.391
5.00	58.441	1.151	56.184	60.697	72.000	1.544	68.974
Overall	64.433	.433	63.585	65.282	72.000	1.325	69.402

Group 1= Acetone (Control); Group 2= 0.25 µL/mL air; Group 3= 0.50 µL/mL air; Group 4= 0.75 µL/mL air; Group 5= 1.0 µL/mL air; [a]Estimation is limited to the largest survival time if it is censored.

Supplementary Table S4. Overall comparisons of survival of *A. pongamiae* exposed to different concentrations of AVEO over time Log Rank (Mantel-Cox) test after 24, 48, and 72 h in fumigant toxicity test

	Chi-Square	Df	Sig.
Log Rank (Mantel-Cox)	182.167	4	<0.001

Supplementary Table S5. Percentage mortality of *A. pongamiae* in contact toxicity test after 24, 48, and 72 h. exposure of AVEO.

Concentration	24 h	48 h	72 h
Acetone (Negative control)	2.22	6.67	10
2.5 µL/mL	4.44	18.89	35.56
5 µL/mL	8.89	26.67	51.69
7.5 µL/mL	15.56	34.44	63.33
10 µL/mL	20	50	67.78
Abamectin (Positive control)	66.67	85.71	99.17

Supplementary Table S6. Case processing summary of contact toxicity test using Kaplan-Meier survival analysis

Group	Total N	N of Events	Censored	
			N	Percent
1.00	270	17	253	93.7%
2.00	270	53	217	80.4%
3.00	270	78	192	71.1%
4.00	270	101	169	62.6%
5.00	270	124	146	54.1%
6.00	270	226	44	16.3%
Overall	1620	599	1021	63.0%

Group 1= Acetone (Negative control); Group 2= 2.50 µL/mL; Group 3= 5.0 µL/mL; Group 4= 7.50 µL/mL; Group 5= 10 µL/mL; Group 6= Abamectin (Positive control). [a]Estimation is limited to the largest survival time if it is censored.

Supplementary Table S7. Means and medians for survival time in contact toxicity test using Kaplan-Meier survival analysis.

Group	Mean[a]				Median		
	Estimate	Std. Error	95% Confidence Interval		Estimate	Std. Error	95% Confidence Interval
			Lower Bound	Upper Bound			Lower Bound
1.00	70.850	.415	70.037	71.664	.	.	.
2.00	69.056	.621	67.838	70.273	.	.	.
3.00	67.473	.754	65.994	68.951	72.000	2.575	66.953
4.00	65.755	.864	64.060	67.449	72.000	1.991	68.097
5.00	63.200	.968	61.303	65.097	72.000	1.822	68.430
6.00	53.244	1.177	50.937	55.552	48.000	2.286	43.519
Overall	64.712	.377	63.974	65.450	72.000	1.002	70.037

Group 1= Acetone (Negative control); Group 2= 2.50 µL/mL; Group 3= 5.0 µL/mL; Group 4= 7.50 µL/mL; Group 5= 10 µL/mL; Group 6= Abamectin (Positive control). [a]Estimation is limited to the largest survival time if it is censored.

Supplementary Table S8. Overall comparisons of survival of *A. pongamiae* exposed to different concentrations of AVEO over time Log Rank (Mantel-Cox) test after 24, 48, and 72 h in contact toxicity test.

	Chi-Square	df	Sig.
Log Rank (Mantel-Cox)	394.201	5	<0.001

Test of equality of survival distributions for the different levels of Group.
